# Porcine circovirus type 2 exploits JNK-mediated disruption of tight junctions to facilitate *Streptococcus suis* translocation across the tracheal epithelium

**DOI:** 10.1186/s13567-020-00756-2

**Published:** 2020-02-27

**Authors:** Qing Wang, Hong Zhou, Huixing Lin, Zhe Ma, Hongjie Fan

**Affiliations:** 1 MOE Joint International Research Laboratory of Animal Health and Food Safety, College of Veterinary Medicine, Nanjing Agricultural University, Nanjing, China; 2 Jiangsu Co-innovation Center for Prevention and Control of Important Animal Infectious Diseases and Zoonoses, Yangzhou, China

## Abstract

Porcine circovirus type 2 (PCV2) is considered as the primary pathogen of porcine circovirus-associated disease (PCVAD), which results in significant economic losses worldwide. Clinically, PCV2 often causes disease through coinfection with other bacterial pathogens, including *Streptococcus suis* (*S. suis*), and especially the highly prevalent *S. suis* serotype 2 (SS2). The present study determined that continuous PCV2 infection in piglets down-regulates tight junction proteins (TJ) ZO-1 and occludin in the lungs. Swine tracheal epithelial cells (STEC) were used to explore the mechanisms and consequences of disruption of TJ, and an in vitro tracheal epithelial barrier model was established. Our results show that PCV2 infection in STEC decreases the expression levels of ZO-1 and occludin and increases the permeability of the tracheal epithelial barrier, resulting in easier translocation of SS2. Moreover, Western blot analysis indicates that PCV2 infection activates the JNK/MAPK pathway. The disruption of TJ in SETC and increased permeability of the epithelial barrier induced by PCV2 could be alleviated by inhibition of JNK phosphorylation, which indicates that the JNK/MAPK pathway regulates the expression of ZO-1 and occludin during PCV2 infection. This study allows us to better understand the mechanisms of PCV2 coinfection with bacterial pathogens and provides new insight into controlling the occurrence of PCVAD.

## Introduction

Porcine circovirus type 2 (PCV2) is highly prevalent worldwide. It is the primary causative agent of porcine circovirus-associated disease (PCVAD), a disease associated with postweaning multisystemic wasting syndrome, reproductive disorders, enteric diseases and respiratory signs, which are responsible for great economic losses [1]. Horizontal transmission via the respiratory tract is one of the main routes of PCV2 infection [2]. *Streptococcus suis* (*S. suis*) is considered to be a commensal colonizer of the upper respiratory tract in swine, but it can also breach the epithelial barriers and cause infections, leading to arthritis, meningitis, endocarditis, pneumonia and septicemia [3, 4]. A total of 35 serotypes have been reported based on capsular polysaccharide antigens, and serotype 2 (SS2) is frequently isolated from clinical diseased pigs [5]. In clinical studies, coinfections can often be determined among PCVAD cases [6]. The morbidity and mortality of PCVAD increases when PCV2 affected pig herds experience secondary infections or concurrent infections with other pathogens, such as *Mycoplasma hyopneumoniae* [6, 7]. In recent years, PCV2 and *S. suis* coinfection cases have been frequently detected [8]; however, there is poor understanding of whether/how PCV2 increases the risk of infection with *S. suis*.

Viruses predispose the host to infection by bacterial pathogens by various mechanisms, including disruption of the epithelium barrier, upregulation of adhesion proteins and suppression of the immune response [9]. Both PCV2 and SS2 are pathogens that can cause disease through infection of the respiratory tract; thus, we hypothesized that PCV2 could promote SS2 translocation across the respiratory epithelial barrier. The respiratory epithelium is an important barrier to defend against foreign particles; dysfunction of the epithelial barrier is an important cause of pathogen invasions [10]. The integrity of the epithelial barrier is maintained by intercellular junctional complexes, including tight junctions, adherens junctions and desmosomes [11, 12]. Tight junction proteins (TJ) are located at the apicolateral boundary of epithelial cells, and they significantly contribute to the barrier function of the epithelium [12]. However, the integrity of TJ could be disrupted during infection, resulting in the passage of microbial pathogens into subepithelial tissues [13]. The aim of the study is to investigate whether and how PCV2 infection disrupts TJ and contributes to SS2 translocation.

Numerous signaling pathways have been reported to be involved in the assembly, disassembly, and maintenance of TJ; these include protein kinase C [14], Rho GTPase [15], myosin light chain kinase [16] and mitogen-activated protein kinase (MAPK) signaling pathways [17]. The MAPK pathway is able to regulate the expression of TJ in different epithelial cells, and the most extensive families of MAPK include p38, ERK1/2, and JNK [18–21].

In this study, the effects of PCV2 on expression levels of TJ were assessed in vivo and in vitro. An in vitro tracheal epithelial barrier model was constructed, and we compared the number of SS2 colonies penetrating across the uninfected and PCV2-infected epithelial barriers and assessed the associated changes in paracellular permeability. In addition, the roles of the involvement of the MAPK pathway in TJ in PCV2-infected cells were explored.

## Materials and methods

### Bacteria, virus and cell lines

*Streptococcus suis* serotype 2 (SS2) strain *ZY05719* is a virulent strain which was isolated from a diseased pig in Sichuan, China in 2005. SS2 *ZY05719* was cultured on Todd-Hewitt agar (THA, BD, USA) overnight, and colonies were then isolated and inoculated in Todd-Hewitt broth (THB, BD, USA) medium; these were then incubated to Logarithmic growth phase.

The PCV2 strain used in this study was isolated from Anhui, China. Virus stock was prepared in a 20-passage cell culture of PK-15 cells with a titer of 10^6.5^ TCID_50_/mL.

Immortalized swine tracheal epithelial cells (STEC) were cultured in Dulbecco Modified Eagle medium (DMEM, Gibco, USA) supplemented with 10% (v/v) fetal bovine serum (FBS, Gibco, USA) at 37 °C in 5% CO_2_. For assays, STEC were digested with trypsin, suspended in culture medium, distributed into 24-well cell culture plates, and incubated until the cells were confluent.

### Animal experiments

Three-week-old, Duroc × Long White × Large White, crossbred piglets were selected from a healthy pig farm, the piglets were porcine-colostrum-deprived and non-immunised. All piglets were confirmed seronegative for PCV2, porcine reproductive and respiratory syndrome virus (PRRSV), classical swine fever virus (CSFV), *Haemophilus parasuis* (HPS) and SS2 by commercial ELISA detection kits (PCV2, PRRSV, CSFV and SS2 antibody test kits, Keqian, China; HPS antibody test kit, Biovet, Canada) according to the manufacturer’s instructions. One week later, piglets were randomly divided into 2 groups consisting of the control group (2 piglets) and the PCV2-infected group (3 piglets). Piglets were infected with PCV2 through intranasal (2 mL) and intramuscular (3 mL) inoculation or inoculated with DMEM through the same route. The infective doses and inoculation methods were determined by consulting the literature [22, 23]. Blood samples were taken at 0, 5, 8, 11, 14, 17, 21, 24 and 28 days post-inoculation (dpi) with PCV2 for serum isolation. The contents of PCV2 in sera were evaluated through absolute qRT-PCR assay with forward primer 5′-GGCTCCACTGCTGTTATTCT-3′ and reverse primer 5′-TAGGAGAAGGGCTGGGTTAT-3′. Viral loads test was performed in triplicate for each sample and each set of qRT-PCR assay was repeated three times. All the piglets were sacrificed and necropsied at 28 dpi with PCV2. The lung samples (apical, middle, caudal right lobe and accessory lobe) were collected for further analysis. The total protein and RNA extraction samples were prepared from taking equal amounts of lung tissue from different areas of the lungs. The animal experiments were approved by the Ethical Committee for Animal Experiments of the Nanjing Agricultural University (Protocol number: PT-027) and were in accordance with the guidelines of the Animal Welfare Council of China.

### Immunofluorescence staining of ZO-1 and occludin proteins

The right middle lung lobes from piglets were cut and fixed with 10% neutral buffered formalin (Solarbio, Beijing, China). The tissues were embedded in paraffin wax and cut into 5 μm thick sections. After the dewaxing process and antigen repairment as previously described [24], the slides were blocked with 2% BSA (Solarbio, Beijing, China). For Immunofluorescence staining of ZO-1 and occludin in STEC, the cells were fixed with 4% Paraformaldehyde (Biosharp Life Science, China) for 15 min before being blocked with 2% BSA for 1 h at 37 °C. Samples were incubated with primary antibodies ZO-1 (5 µg/mL, Invitrogen™, USA) and occludin (5 µg/mL, Invitrogen™, USA) at 4 °C overnight. Then, samples were incubated with secondary antibody (Goat anti-mouse IgG, AF594, 5 µg/mL, Invitrogen™, USA) for 50 min at room temperature. Nuclei were stained by DAPI (Solarbio, Beijing, China). Finally, all the specimens were examined with a Zeiss laser scanning microscope (Carl Zeiss, Germany).

### Infection of PCV2 in STEC

PCV2 was inoculated onto confluent STEC monolayers at a multiplicity of infection (MOI) of 1 for 24 h, 48 h and 72 h as described [25], to confirm infection, indirect fluorescence assays were performed as described [26] with some modifications. Briefly, cells were fixed with methanol for 30 min at 4 °C, then incubated with PCV2 capsid protein mouse monoclonal antibody (generated in our lab) at 37 °C for 1 h. After washing with PBST (PBS containing 0.1% Tween 20), the samples were incubated with DyLight488-conjugated goat anti-mouse IgG antibody (Abbkine, China) at 37 °C for 45 min. Finally, the cells were stained 5 min with DAPI (Solarbio, Beijing, China) and examined under a fluorescence microscope (Zeiss, Germany). In addition, STEC were infected with PCV2 for 12 h, 24 h, 36 h and 48 h as described above; viral DNA were extracted from supernatants and cells using a Viral DNA Kit (OMEGA, USA). PCV2 proliferation in STEC was determined through absolute qRT-PCR assay with primers described above. Assays were performed as three independent experiments and each set of qPCR assay was repeated three times.

### Adherence and invasion assays

Uninfected and PCV2 infected STEC were grown in 24-well plates for 24 h, 36 h or 48 h. SS2 strain *ZY05719* was cultured in THB until OD_600_ reached 0.7. Bacteria were collected by centrifugation at 5000 rpm for 5 min, washed three times with phosphate buffer saline (PBS), and appropriately diluted in DMEM without FBS. Both uninfected and PCV2 infected STEC were infected with *ZY05719* with an MOI of 10 (5 × 10^6^ CFU/well) [27]. The cell plates were centrifuged at 800 × *g* for 10 min. After 2 h of incubation at 37 °C with 5% CO_2_, the cells were washed with PBS to remove non-adherent bacteria. For the adhesion assay, the cells were lysed with double-distilled water. The number of adherent bacteria was determined by spreading on THA. For invasion assay, extracellular bacteria were killed by the addition of DMEM containing 5 μg/mL penicillin (Solarbio, Beijing, China) and 100 µg/mL gentamicin (Solarbio, Beijing, China); the cells were then incubated for another hour. Antibiotic treated cells were washed three times in PBS, lysed with double-distilled water, and spread on THA to determine bacterial counts. The adherent and invasive bacteria was compared with the input number (5 × 10^6^ CFU/well), and the adhesion or invasion rate was calculated as (CFU that adherent or invasive to STEC/5 × 10^6^ CFU) × 100%. The adhesion and invasion rates of the SS2-infected alone group were normalized to 100%. Assays were performed as five separate experiments.

### Construction of an in vitro tracheal epithelial barrier model and translocation of SS2

SETC (1 × 10^5^ cells) were plated on the top side of polytetrafluoroethylene 3 μM pore-size membrane Transwells (Corning, USA). Culture medium was changed every other day until stable electrical resistance was attained. The transepithelial electrical resistance (TEER) was measured with the Millicell ERS-2 electrical resistance system (Millipore, USA).

Experiments were performed when STEC monolayers reached a steady state. Transwells containing STEC were either uninfected, infected with PCV2 for 36 h or for 48 h before inoculation with SS2 strain *ZY05719* (1.5 × 10^7^ CFU in 500 μL DMEM) and 2 h incubation (as determined in preliminary experiments). Four transwells were used per group. The TEER was measured and is shown relative to the TEER at the initial time point (before infection). The relative TEER of the control group was normalized to 100%. The medium in the lower chamber of the transwells was collected and spread on THA plates to count bacterial colonies. Assays were repeated as three independent experiments.

### Western blotting

Total protein of the lung tissues from piglets were extracted with Whole Cell Lysis Assay (KeyGEN Biotech, Nanjing, China) according to the instructions. Briefly, the tissues were cut into pieces and lysed in a cold lysis buffer, centrifuged after homogenization, and the supernatant was collected. STEC were lysed with RIPA plus 1 mM PMSF (KeyGEN Biotech, Nanjing, China), centrifuged and the supernatant was collected. The protein concentrations were determined using a Pierce™ BCA Protein Assay Kit (Thermo Fisher Scientific, USA). SDS-PAGE Sample Loading Buffer (KeyGEN Biotech, Nanjing, China) was added to the lysate protein and boiled for 10 min. Then, equal amounts of protein for each sample was separated on a gel by SDS-PAGE and transferred onto 0.22 μm polyvinylidene fluoride (PVDF) membrane (Millipore, USA). The membranes were blocked in 5% non-fat milk for 2 h at 37 °C, and incubated with anti-ZO-1 mouse mAb (1 µg/mL, Invitrogen™, USA), anti-occludin mouse mAb (1 µg/mL, Invitrogen™, USA) and anti-GAPDH mouse mAb (1:5000, CMCTAG, USA) at 4 °C overnight. Subsequently, the membranes were incubated with secondary antibody HRP-goat anti-mouse IgG H&L (1:5000, CMCTAG, USA) for 1 h at 37 °C. Detection of protein bands was performed using ECL Pico-Detect™ Western Blotting Substrate (CMCTAG, USA) according to the manufacturer’s instructions. For further exploration of MAPK pathway activation, the primary and secondary antibodies used were as follows: phospho-p38 rabbit mAb and p38 rabbit mAb, phospho-ERK rabbit mAb and ERK rabbit mAb, phospho-JNK rabbit mAb and JNK rabbit mAb (1:1000, Cell Signaling Technology, USA), anti-GAPDH rabbit mAb (1:5000, CMCTAG, USA) and HRP-goat anti-rabbit IgG H&L (1:5000, CMCTAG, USA). Band intensities were analyzed by Image J software and the band intensities of the control group at each time point were normalized to 1.0.

### qRT-PCR

To determine transcript levels of ZO-1 and occludin, total RNA was extracted from lung tissues using Total RNA Kit I (OMEGA, USA) and STEC using TRIzol reagent (Takara, Japan). Reverse transcriptase reactions were performed using the HiScript Q RT SuperMix for qPCR (+gDNA wiper) (Vazyme, Nanjing, China) according to the manufacture’s instruction. Quantitative Real-Time Polymerase Chain Reaction (qRT-PCR) was performed on the 7300 Real-Time PCR System (Applied Biosystems, USA) with SYBR^®^ Premix Ex Taq™ II (Tli RNaseH Plus) (Takara, Japan). The primers used are listed in Table 1. The *GAPDH* gene was used as an internal control, and relative quantification compared to the uninfected cells was calculated based on the 2^−ΔΔCt^ method [28]. The tests were performed in triplicate and each set of qPCR assay was repeated three times.

**Table 1:** **List of qRT-PCR primers**

Gene	Forward	Reverse
GAPDH	GATGCTGGTGCTGAGTATGT	GGCAGAGATGATGACCCTTT
occludin	CGGATTCTGTCTATGCTCGTTAT	TAGCCCATACCACCTCCTATT
ZO-1	GGGTGTTGAGCTCCATAGAAA	GTCTCGGCAGACCTTGAAATA

### Inhibition assay

To inhibit the activity of JNK, 10 μM JNK/MAPK inhibitor SP600125 (dissolved in DMSO, MCE, USA) was added into PCV2 viral stock in advance [29], and equal amounts of DMSO (Solarbio, Beijing, China) were added into PCV2 viral stock and DMEM as controls. Confluent STEC were mock-infected or infected with PCV2 for 36 h or 48 h.

### Analysis of paracellular permeability

The paracellular permeability of the tracheal epithelial barrier was measured using FITC-conjugated dextran 4000 (Sigma, USA). STEC were cultured in Transwell inserts as described above. Four transwells were used per group. After experimental treatments, the media in the upper and lower chambers were removed; the lower chamber was refilled with fresh culture medium DMEM and the upper chamber was refilled with DMEM containing 1 mg/mL FITC-conjugated dextran (4 kDa). After 1 h, 100 μL of sample was collected from the lower chamber and the fluorescence was detected with Tecan Infinite 200 PRO. Paracellular permeability was shown relative to permeability of control monolayers. Assays were repeated as three independent experiments.

### Statistical analysis

The results in the study were analyzed and graphed using software SPSS Statistics 17.0 and GraphPad Prism 5. Statistical significant differences were assessed using Student *t* test and one-way analysis of variance (ANOVA) with 95% confidence intervals. A *P* value less than 0.05 was considered to be significant.

## Results

### Continuous infection with PCV2 caused down-regulation of tight junction proteins in the lungs of piglets

**Figure 1:**
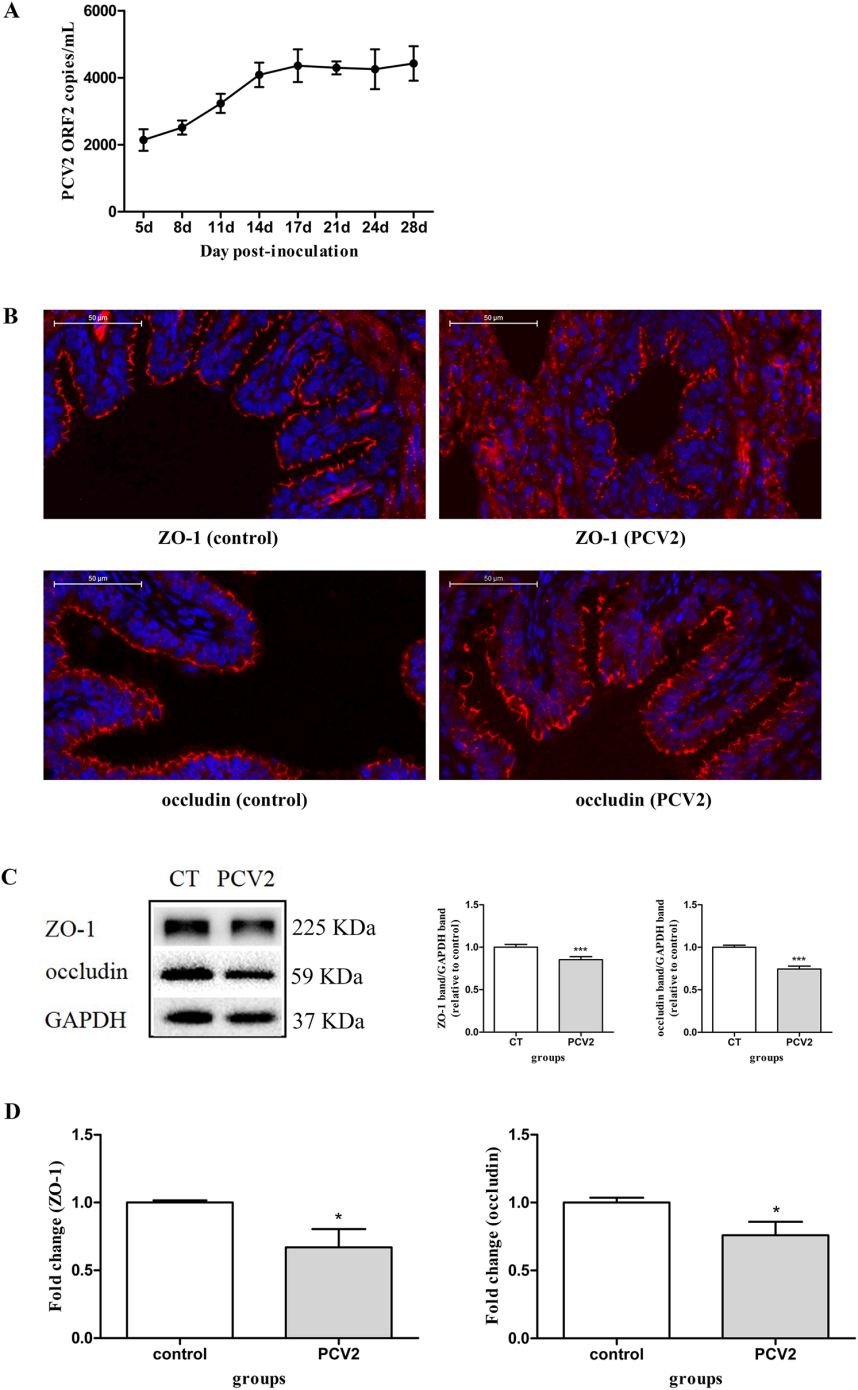
**PCV2 down-regulates tight junction protein levels in the lungs of piglets. A** Viral loads in the serum of the PCV2-infected piglets. The piglets were continuously infected with PCV2. **B** Immunofluorescence staining of ZO-1 and occludin in the lungs. Scale bar, 50 μm. **C** Protein levels of ZO-1 and occludin in the lungs were determined by Western blotting and band intensities relative to control were analyzed. CT, the control group. **D** qRT-PCR analysis of ZO-1 and occludin in the lungs. The results are shown as mean ± SD of three times experiments. Significant differences were determined using Student *t*-test. *P < 0.05. **P < 0.01; ***P < 0.001.

To assess the effect of PCV2 infection on TJ of the host respiratory epithelia, piglets were uninfected or infected with PCV2. The PCV2 loads in serum were detected by qRT-PCR assay, PCV2 infection induced increased viral serum loads at 17 dpi and subsequently exhibited persistent infection during the experiments (Figure 1A). We could not detect PCV2 in the control group and on 0 dpi in all piglets, indicating good primer specificity. The piglets were slaughtered at 28 dpi, at which time the piglets had been infected for a period of time. The effects of PCV2 infection on TJ in the lungs were determined by immunofluorescence, Western blotting and qRT-PCR assays. As shown in Figure 1B (or Additional file 1) and C, ZO-1 and occludin in PCV2-infected group were disrupted and the protein levels were reduced compared with the control group. In addition, the mRNA levels of ZO-1 and occludin in the lungs from the PCV2 infected group were also lower than those from the control group (Figure 1D). The results suggest that PCV2 infection down-regulates the expression of TJ in the lungs.

### Infection of PCV2 in STEC

**Figure 2:**
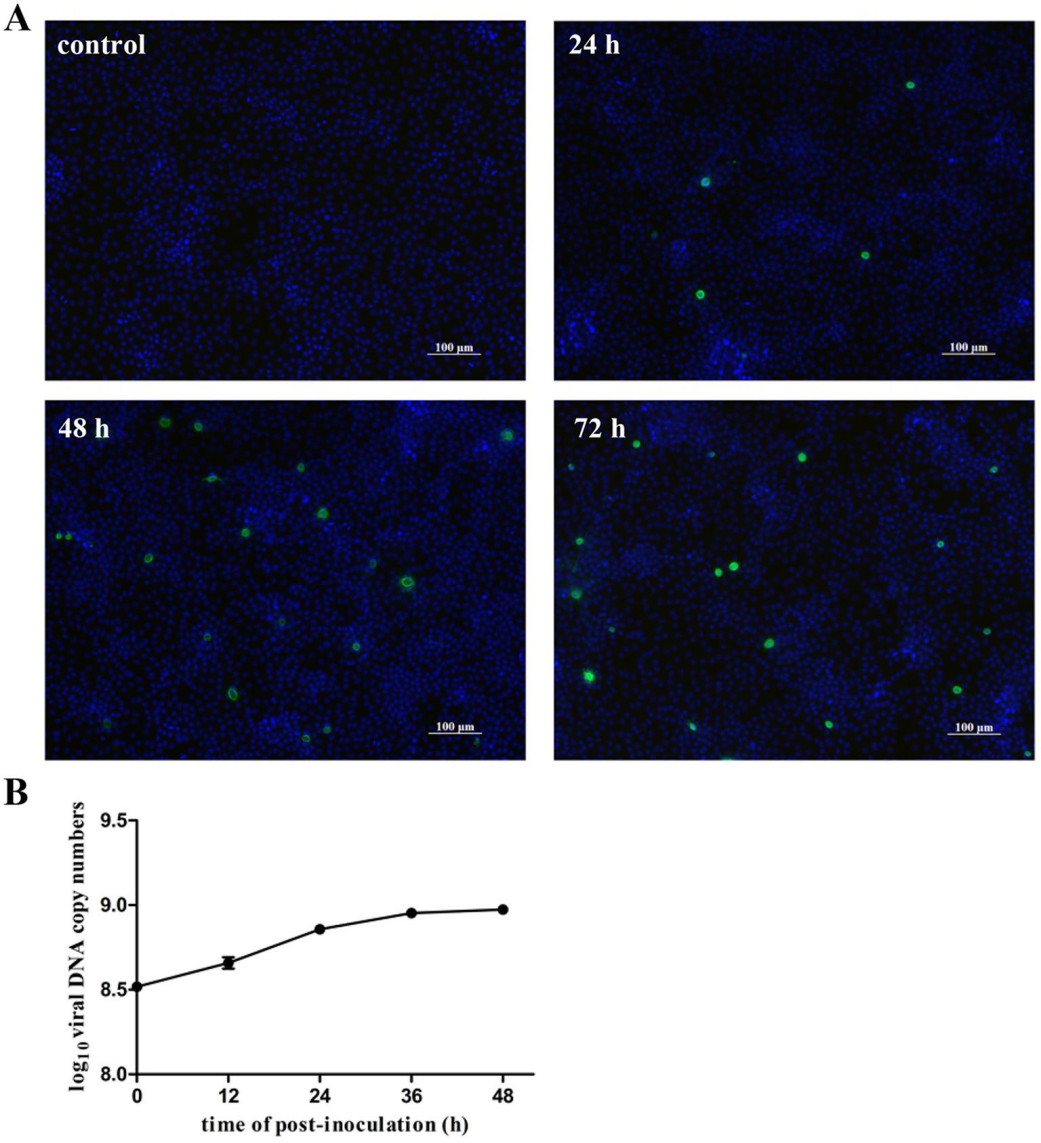
**PCV2 infection in STEC. A** Detection of PCV2 infection in STEC by indirect immunofluorescence assay. Green fluorescence indicates that cells were infected with PCV2. Scale bar, 100 μm. **B** Proliferation of PCV2 in STEC. The cells were infected with PCV2 at MOI 1, the supernatants and cells were frozen and thawed 3 times at 12 h, 24 h, 36 h and 48 h post-infection. Viral DNA were extracted and quantitated by qRT-PCR assay. The results are shown as mean ± SD of three separate experiments.

To establish an in vitro PCV2-infected cell model, PCV2 infection in STEC were examined by immunofluorescence assays. PCV2 infection could be detected at 24 h post-infection (Figure 2A). To confirm the proliferation of PCV2 in cells, viral DNA were extracted and detected by qRT-PCR; viral DNA increased from 12 h to 48 h (Figure 2B). These results indicate that PCV2 can infect and replicate in STEC.

### Effects of PCV2 infection on adherence and invasion of SS2

**Figure 3:**
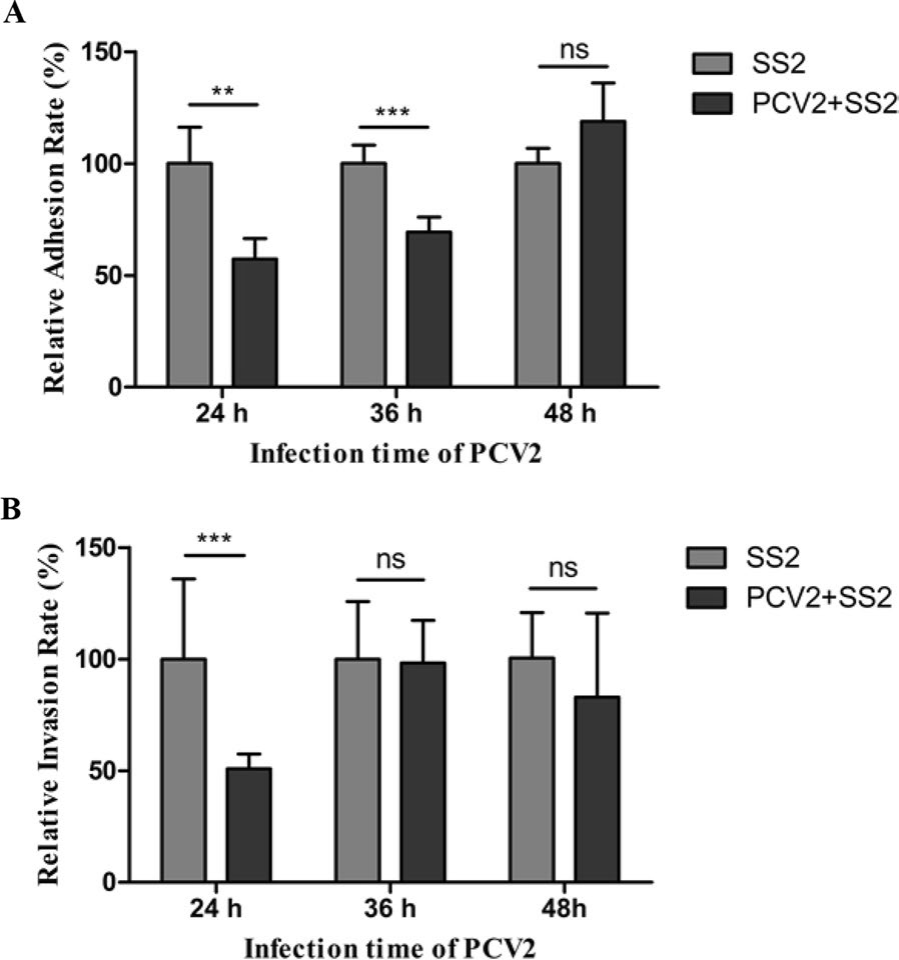
**Adherence and invasion assays.** STEC were uninfected or infected with PCV2 for 24 h, 36 h and 48 h before incubating with SS2 at MOI 10. The adherent and invasive bacteria was compared with the input number. **A** Rates of SS2 adherence to STEC. **B** The invasion rates of SS2 recovered from invaded STEC. The results are shown as mean ± SD of five independent experiments. Significant differences were determined using Student *t*-test. **P < 0.01; ***P < 0.001; ns: not significant.

STEC were pre-infected with PCV2 for 24 h, 36 h or 48 h before incubation with SS2 strain *ZY05719*. Bacterial adherence and invasion experiments were performed. As shown in Figure 3A and Additional file 2, fewer SS2 adhered to infected STEC than control STEC when the cells were pre-infected with PCV2 for 24 h and 36 h. When the cells were infected with PCV2 for 48 h, no differences in SS2 adherence were observed. In contrast with control cells, the numbers of bacteria that invaded STEC pre-infected with PCV2 for 24 h were significantly lower; however, the numbers of invasive bacteria were not significantly different among treatments with prolonged PCV2 infection times of 36 h and 48 h (Figure 3B and Additional file 3).

### Infection of PCV2 increased permeability and contributed to SS2 translocation across the tracheal epithelial barrier

**Figure 4:**
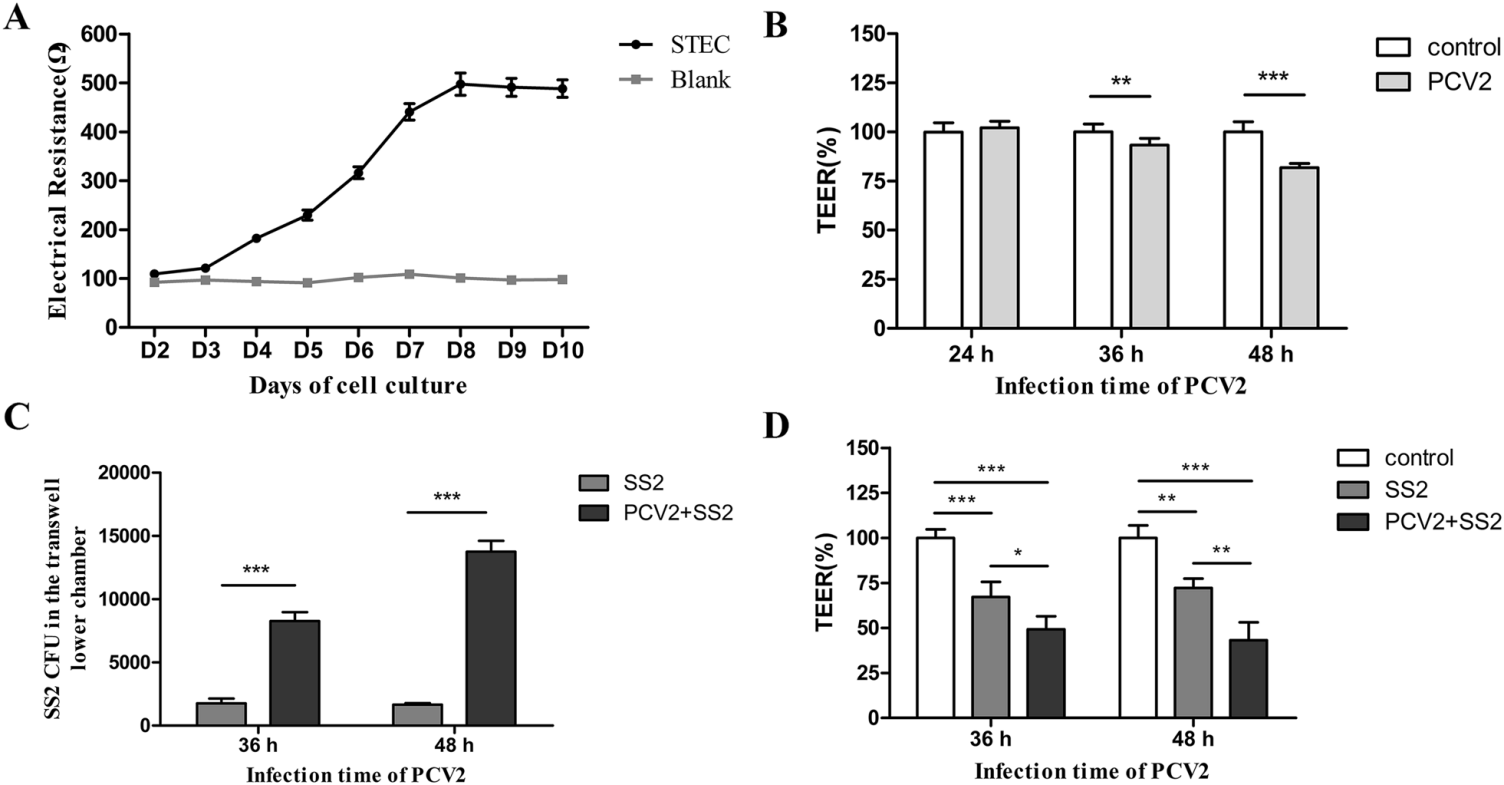
**PCV2 infection increased barrier permeability and SS2 translocation. A** Construction of an epithelial barrier model in vitro using STEC. The electrical resistance increased from day 2 to day 8 and formed tight cellular connections. **B** PCV2 infection decreased the TEER and increased the permeability of the epithelial barrier. STEC monolayers were infected with PCV2 for 24 h, 36 h and 48 h, TEER was measured and was shown relative to the TEER before infection. **C** PCV2 infection contributed to translocation of SS2 across the tracheal epithelial barrier. The in vitro epithelial barriers were infected with PCV2 for 36 h or 48 h before incubation with SS2 for 2 h, and the number of SS2 penetrating across the epithelial barrier model were counted. **D** Coinfection increased the barrier permeability compared with the SS2 infection alone. STEC monolayers were uninfected or infected with PCV2 for 36 h or 48 h before incubation with SS2 for 2 h, TEER was measured and was shown relative to the TEER before infection. The results are described as mean ± SD of three independent experiments. Significant differences in **B** and **C** were determined using the Student *t*-test. Significant differences in **D** were determined using one-way ANOVA analysis. *P < 0.05; **P < 0.01; ***P < 0.001.

To gain further insights into the consequences of the ZO-1 and occludin down-regulation we observed in vivo, we constructed an in vitro tracheal epithelial barrier model with the STEC. The TEER increased rapidly during the initial 7 days, reaching a stable level around day 8 and this level was maintained for the next 3 days (Figure 4A). The in vitro epithelial barrier models were infected with PCV2 at day 8. Compared with the control group, PCV2 infection significantly decreased the TEER at 36 h and 48 h after infection, and there was no differences at 24 h post-infection of PCV2 (Figure 4B). Measuring of the TEER has been widely accepted as a quantitative technique for monitoring epithelial barrier integrity [30]. Then, the numbers of SS2 penetrating across the epithelial barrier model were counted after 36 h and 48 h of PCV2 infection with SS2 infection times of 2 h. The number of translocated SS2 in the PCV2 infected group was greater than that in the control group (Figure 4C). Furthermore, compared to the SS2-infected alone, PCV2 and SS2 coinfection resulted in a greater TEER reduction (Figure 4D), indicating that coinfection increased the permeability of the epithelial barrier. Thus, we established an in vitro tracheal epithelial barrier model using STEC; PCV2 infection gradually reduced the TEER value and induced breakdown of the epithelial barrier integrity; PCV2 and SS2 coinfection induced increased-permeability and more SS2 translocation compared to SS2 infection alone.

### Continuous infection with PCV2 reduced the expression of tight junction proteins in STEC

**Figure 5:**
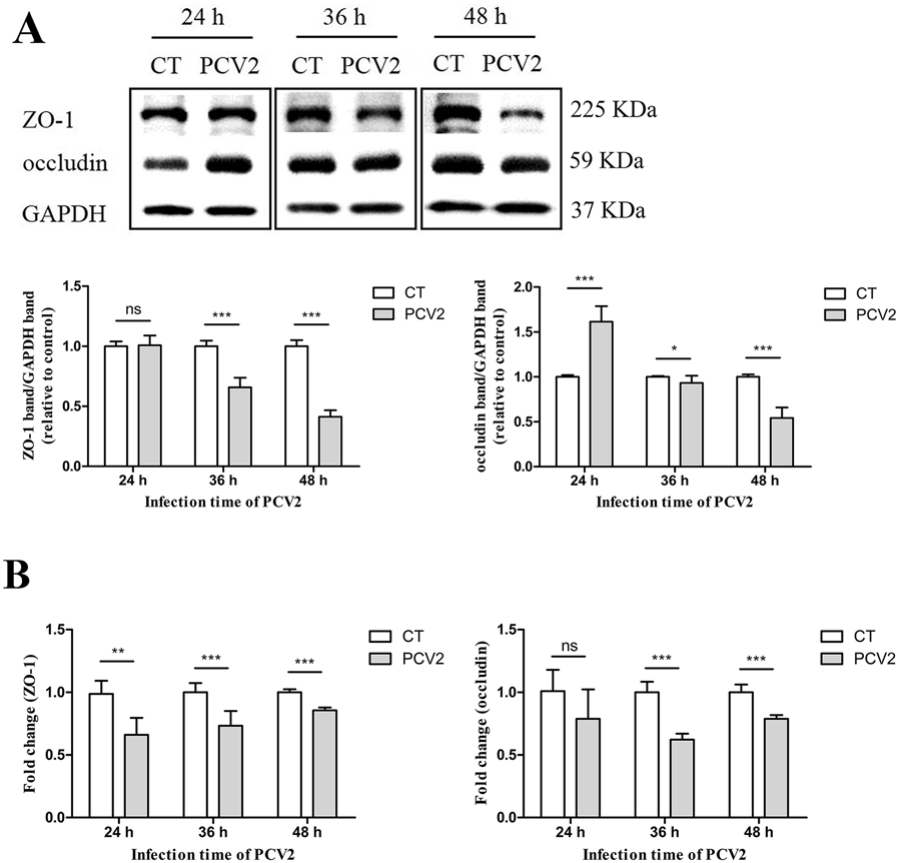
**PCV2 down-regulates tight junction protein levels in STEC.** STEC were uninfected or infected with PCV2 for 24 h, 36 h and 48 h. **A** Protein levels of ZO-1 and occludin were determined by Western blotting and band intensities relative to control were analyzed. **B** mRNA levels of ZO-1 and occludin were analyzed with qRT-PCR. The results are described as mean ± SD of three independent experiments. Significant differences were determined using the Student *t*-test. *P < 0.05; **P < 0.01; ***P < 0.001; ns: not significant.

To investigate the effect of PCV2 on tight junction proteins of STEC, cells were incubated with PCV2 for 24 h, 36 h and 48 h. The cell proteins were then collected for Western blot and total RNA were extracted for qRT-PCR analysis. Western blot analysis shows that STEC infected with PCV2 for 48 h significantly reduced levels of tight junction proteins ZO-1 and occludin (Figure 5A). Moreover, the qRT-PCR results demonstrate that PCV2 down-regulated the mRNA levels of ZO-1 and occludin (Figure 5B). These results suggest that PCV2 continuous infection in STEC would cause downregulation of tight junction protein levels.

### Coinfection with PCV2 and SS2 reduced tight junction protein levels in STEC

**Figure 6:**
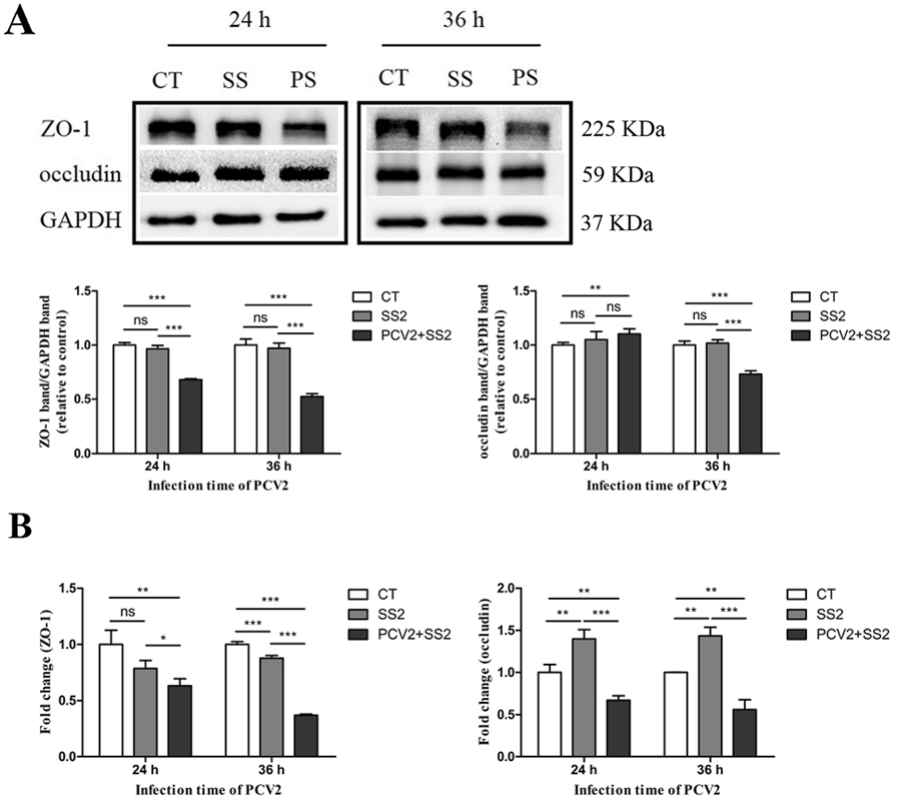
**Coinfection with PCV2 and SS2 causes down-regulation of TJ in STEC.** STEC were infected with PCV2 for 24 h or 36 h before incubating with SS2 for 12 h. **A** Protein levels of ZO-1 and occludin were determined by Western blotting and band intensities relative to control were analyzed. **B** mRNA levels of ZO-1 and occludin were analyzed with qRT-PCR. The results were described as mean ± SD of three independent experiments. Significant differences were determined using one-way ANOVA analysis. *P < 0.05; **P < 0.01; ***P < 0.001; ns: not significant.

Uninfected control STEC and those infected with PCV2 for 24 h and 36 h were incubated with SS2 for another 12 h. For the coinfected cells, the total infection time of PCV2 were 36 h and 48 h, at the time points, PCV2 infection induced a decrease in the TJ levels (Figure 5). The infection time of SS2 was based on the results in preliminary experiments, the protein levels of ZO-1 and occludin in STEC infected with SS2 for 4, 8 and 12 h were not reduced, while SS2 infection could affect the distribution of TJ (these data have not been published). As shown in Figure 6A, B, both Western blot and qRT-PCR results show that coinfection induced significantly lower expression levels of ZO-1 and occludin than those in SS2 single-infected cells. However, SS2 infection did not cause reduction of ZO-1 and occludin protein levels compared with the control group (Figure 6A). These results indicate that PCV2 and SS2 coinfection lead to disruption of ZO-1 and occludin proteins, and the destructive effects were related to PCV2 pre-infection.

### Activation of JNK/MAPK is required for down-regulation of tight junction proteins and disruption of the epithelial barrier caused by PCV2 infection

**Figure 7:**
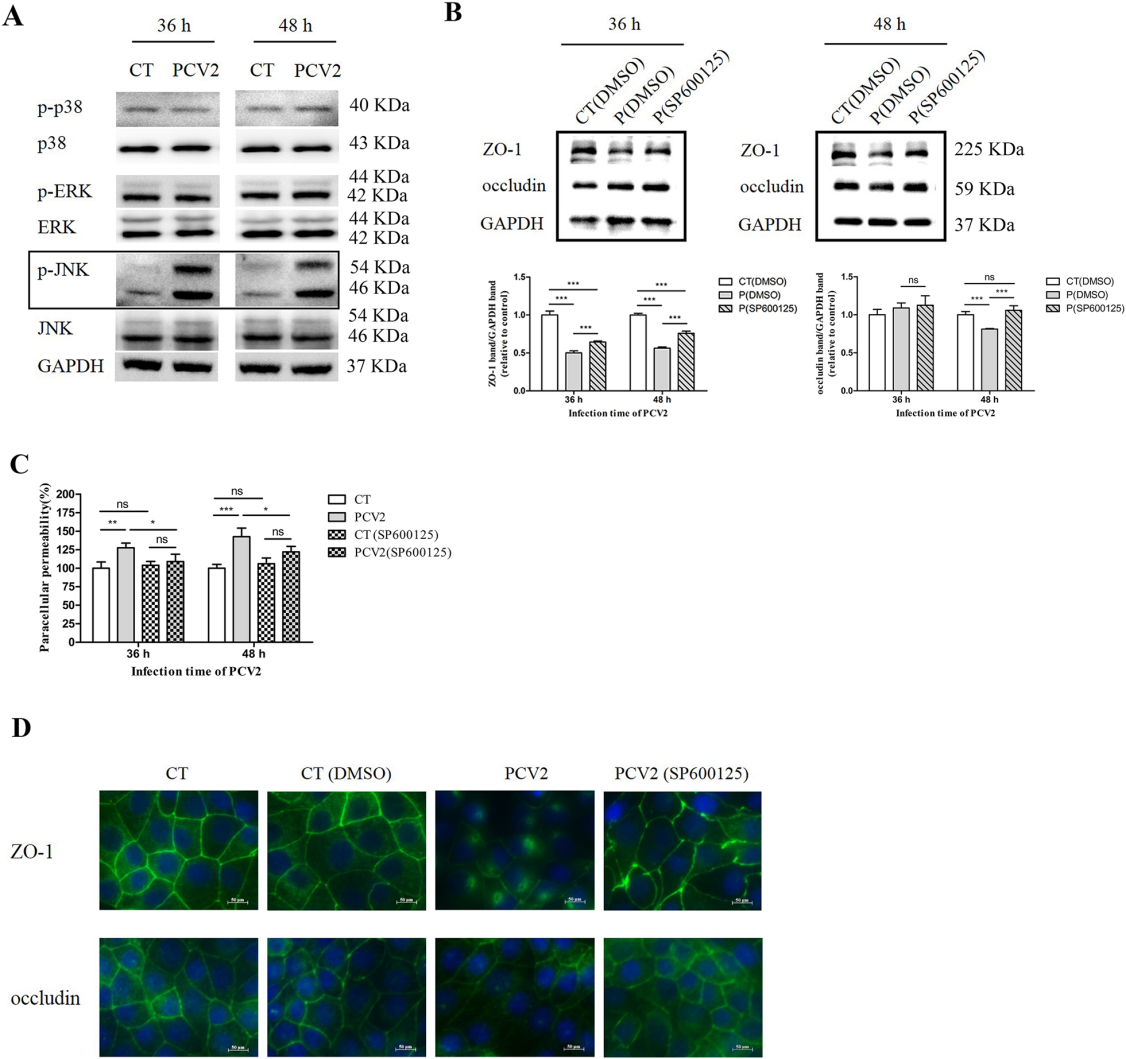
**Activation of JNK/MAPK pathway is related to the disruption of TJ and increased-permeability of tracheal epithelial barrier. A** Western blotting analysis of p38, ERK and JNK phosphorylation in PCV2-infected STEC. STEC were infected with PCV2 for 36 h or 48 h. Phosphorylation of JNK was detected in the process of PCV2 infection. **B** Western blotting analysis of ZO-1 and occludin in PCV2-infected STEC treated with JNK inhibitor SP600125. Band intensities relative to control were analyzed. Compared with the PCV2-infected cells, the disruption of ZO-1 and occludin were alleviated through inhibition of JNK activation. CT, the control group. P, the PCV2 infection group. **C** Measurement of paracellular permeability. PCV2 infection increased the permeability of the epithelial barrier, which can be partially offset by JNK inhibitor. The results are described as mean ± SD of three independent experiments. Significant differences in (B) and (C) were determined using one-way ANOVA analysis. *P < 0.05; **P < 0.01; ***P < 0.001; ns, not significant. **D** Immunofluorescence staining of ZO-1 and occludin in STEC. Scale bar, 50 μm. The infection time of PCV2 was 48 h.

To detect whether the MAPK pathways regulate PCV2-induced disruption of tight junction proteins, STEC were infected with PCV2 for 36 h and 48 h (at the time points, PCV2 infection induced a decrease in the TJ levels); the phosphorylation of p38, ERK and JNK were determined by Western blot. The results show that JNK was phosphorylated, indicating that JNK was activated in the process of PCV2 infection in STEC, while activation of p38 and ERK were not detected (Figure 7A). To determine the function of JNK on tight junction proteins, STEC were treated with inhibitor SP600125 to suppress the activation of JNK during the PCV2 infection period. The cell proteins were collected at 36 h and 48 h of PCV2 post-infection. Western blot analysis shows that inhibiting the activation of JNK would lessen the PCV2 induced reduction of ZO-1 and occludin protein levels to some degree (Figure 7B). Furthermore, we analyzed the epithelial barrier integrity by quantifying the amount of FITC-dextran crossing the Transwell chamber. Relative to the uninfected control, we found that the increased paracellular permeability caused by PCV2 infection were abrogated or partially abrogated by the JNK inhibitor (Figure 7C), providing further evidence that PCV2 induced disruption of the epithelial barrier occurs through the JNK/MAPK pathway. As shown in Figure 7D, the integrity of tight junction proteins is protected when treated with JNK inhibitor in PCV2 infected STEC. In summary, PCV2 infection activates the JNK/MAPK pathway; JNK phosphorylation plays an important role in disruption of TJ in STEC, and is related to the increased-permeability of the epithelial barrier caused by PCV2 infection.

## Discussion

TJ are responsible for the integrity and function of the epithelial barrier, and once damaged, the risk of infection with exogenous pathogens will greatly increase; ZO-1 and occludin are known to play a primary role in the maintenance of the epithelial barrier [31, 32]. In the present study, we demonstrate that PCV2 infection down-regulated the expression of TJ ZO-1 and occludin in the respiratory epithelium, increased the barrier permeability and contributed to the translocation of the bacterial pathogen SS2. Similar findings have been reported: rhinovirus causes the loss of ZO-1 in human airway epithelial cells and disrupts epithelial integrity, which promotes the paracellular migration of *H. influenzae* [33]; HIV-1 down-regulates mRNA and protein levels of TJ (claudin 1, 2, 4, occludin and ZO-1) and impairs epithelial barrier integrity allowing bacterial translocation across the epithelial monolayers [34].

Animal challenge experiments show that PCV2 disrupted the integrity and decreased the mRNA and protein levels of TJ in the lungs. In order to study the consequences and mechanisms of PCV2-induced reduction of TJ, we established an in vitro cell infection model and tracheal epithelial barrier model with STEC. Epithelial cells are target cells for PCV2 infection in vivo and are used for culturing, and for studying internalization and infection of PCV2 in vitro [35–37]. Here, Western blot, qRT-PCR and immunofluorescence assays were carried out to assess the effects of PCV2 on ZO-1 and occludin in STEC. The results were consistent with those in vivo that prolonged the infection time of PCV2 to 48 h in STEC. In addition, PCV2 infection reduced the TEER, increased the permeability of the tracheal epithelial barrier and increased the translocation of SS2. PCV2 infection did not increase the adhesion and invasion of SS2 to STEC during the experiment, suggesting that PCV2 promoted bacterial translocation across the epithelial barrier through the paracellular route, rather than the transcellular route. Although SS2 did not reduce protein levels of ZO-1 and occludin, the TJ were reorganized (data not published), which was reported to be associated with the increased paracellular permeability [38] and bacterial translocation [24].

We found that PCV2 activates JNK/MAPK pathway in STEC, and activation of the pathway plays important roles in the disassembly of tight junctions and affects the barrier functions [39, 40]. Here, the inhibition of JNK rescued the PCV2 induced disruption in ZO-1 and occludin and maintained the barrier permeability to a large extent. A previous study showed that the effector of *Salmonella enteritidis* AvrA could stabilize TJ protein ZO-1 and attenuate bacterial invasion via inhibition of the JNK signaling pathway [41]. The downstream genes of the JNK signaling pathway, including AP-1, c-Jun, ATF-2 and ELK-1, are reported to regulate epithelial barrier functions [41–45]; further study is required to determine which downstream molecules in the JNK pathway were activated by PCV2.

In summary, our data have shown that during PCV2 infection, TJ were down-regulated and barrier function was disrupted, resulting in increased bacterial translocation. Furthermore, we demonstrate that the disrupted TJ and increased permeability were related to the JNK/MAPK pathway. There are still, however, some questions needing to be addressed in future research, such as whether coinfection with PCV2 and SS2 will contribute to the translocation of the virion, and whether the impaired epithelial barrier caused by PCV2 facilitates infection of some other bacterial pathogens except *S. suis*. Together, these findings provide new insights into how PCV2 induces secondary bacterial infection, which might be valuable for strategies targeting the control of tight junction damage.

## Supplementary information

**Additional file 1. Immunofluorescence staining of ZO-1 and occludin in the lungs.**

**Additional file 2. CFU number of adherent bacteria to STEC per well. The results are shown as mean ± SD of five independent experiments.**

**Additional file 3. CFU number of invasive bacteria to STEC per well. The results are shown as mean ± SD of five independent experiments.**

## Abbreviations

PCV2: porcine circovirus type 2
PCVAD: porcine circovirus-associated disease
*S. suis*: *Streptococcus suis*
SS2: *S. suis* serotype 2
TJ: tight junction proteins
STEC: swine tracheal epithelial cells
MAPK: mitogen-activated protein kinases
THA: Todd-Hewitt agar
THB: Todd-Hewitt broth
DMEM: Dulbecco’s Modified Eagle medium
FBS: fetal bovine serum
PRRSV: porcine reproductive and respiratory syndrome virus
CSFV: classical swine fever virus
HPS: *Haemophilus parasuis*
dpi: day post-inoculation
PBS: phosphate buffer saline
TEER: the transepithelial electrical resistance
PVDF: polyvinylidene fluoride
SD: standard deviation

## References

[1] Meng XJ (2013). Porcine circovirus type 2 (PCV2): pathogenesis and interaction with the immune system. Annu Rev Anim Biosci.

[2] Madec F, Rose N, Grasland B, Cariolet R, Jestin A (2008). Post-weaning multisystemic wasting syndrome and other PCV2-related problems in pigs: a 12-year experience. Transbound Emerg Dis.

[3] Lun ZR, Wang QP, Chen XG, Li AX, Zhu XQ (2007). Streptococcus suis: an emerging zoonotic pathogen. Lancet Infect Dis.

[4] Segura M, Calzas C, Grenier D, Gottschalk M (2016). Initial steps of the pathogenesis of the infection caused by Streptococcus suis: fighting against nonspecific defenses. FEBS Lett.

[5] Goyette-Desjardins G, Auger JP, Xu J, Segura M, Gottschalk M (2014). Streptococcus suis, an important pig pathogen and emerging zoonotic agent-an update on the worldwide distribution based on serotyping and sequence typing. Emerg Microbes Infect.

[6] Opriessnig T, Halbur PG (2012). Concurrent infections are important for expression of porcine circovirus associated disease. Virus Res.

[7] Ge XN, Wang F, Guo X, Yang HC (2012). Porcine circovirus type 2 and its associated diseases in China. Virus Res.

[8] Dione M, Masembe C, Akol J, Amia W, Kungu J, Lee HS, Wieland B (2018). The importance of on-farm biosecurity: sero-prevalence and risk factors of bacterial and viral pathogens in smallholder pig systems in Uganda. Acta Trop.

[9] Bosch AA, Biesbroek G, Trzcinski K, Sanders EA, Bogaert D (2013). Viral and bacterial interactions in the upper respiratory tract. PLoS Pathog.

[10] Rezaee F, Georas SN (2014). Breaking barriers. New insights into airway epithelial barrier function in health and disease. Am J Respir Cell Mol Biol.

[11] Schneeberger EE, Lynch RD (2004). The tight junction: a multifunctional complex. Am J Physiol Cell Physiol.

[12] Yuksel H, Turkeli A (2017). Airway epithelial barrier dysfunction in the pathogenesis and prognosis of respiratory tract diseases in childhood and adulthood. Tissue Barriers.

[13] Clarke TB, Francella N, Huegel A, Weiser JN (2011). Invasive bacterial pathogens exploit TLR-mediated downregulation of tight junction components to facilitate translocation across the epithelium. Cell Host Microbe.

[14] Jain S, Suzuki T, Seth A, Samak G, Rao R (2011). Protein kinase Czeta phosphorylates occludin and promotes assembly of epithelial tight junctions. Biochem J.

[15] Arnold TR, Stephenson RE, Miller AL (2017). Rho GTPases and actomyosin: partners in regulating epithelial cell–cell junction structure and function. Exp Cell Res.

[16] Nighot M, Al-Sadi R, Guo SH, Rawat M, Nighot P, Watterson MD, Ma TY (2017). Lipopolysaccharide-induced increase in intestinal epithelial tight permeability is mediated by toll-like receptor 4/Myeloid differentiation primary response 88 (MyD88) activation of myosin light chain kinase expression. Am J Pathol.

[17] Peng C, Ding X, Zhu L, He M, Shu Y, Zhang Y, Li Y, Wang X, Feng S, Li J, Wu J (2019). beta-conglycinin-induced intestinal porcine epithelial cell damage via the nuclear factor kappaB/mitogen-activated protein kinase signaling pathway. J Agric Food Chem.

[18] Matter K, Balda MS (2003). Signalling to and from tight junctions. Nat Rev Mol Cell Biol.

[19] Lei S, Cheng T, Guo Y, Li C, Zhang W, Zhi F (2014). Somatostatin ameliorates lipopolysaccharide-induced tight junction damage via the ERK-MAPK pathway in Caco2 cells. Eur J Cell Biol.

[20] Zhai Z, Ni X, Jin C, Ren W, Li J, Deng J, Deng B, Yin Y (2018). Cecropin A modulates tight junction-related protein expression and enhances the barrier function of porcine intestinal epithelial cells by suppressing the MEK/ERK pathway. Int J Mol Sci.

[21] Wang D, Chadha GK, Feygin A, Ivanov AI (2015). F-actin binding protein, anillin, regulates integrity of intercellular junctions in human epithelial cells. Cell Mol Life Sci.

[22] Liu S, Li W, Wang Y, Gu C, Liu X, Charreyre C, Fan S, He Q (2017). Coinfection with Haemophilus parasuis serovar 4 increases the virulence of porcine circovirus type 2 in piglets. Virol J.

[23] Ramamoorthy S, Opriessnig T, Pal N, Huang FF, Meng XJ (2011). Effect of an interferon-stimulated response element (ISRE) mutant of porcine circovirus type 2 (PCV2) on PCV2-induced pathological lesions in a porcine reproductive and respiratory syndrome virus (PRRSV) co-infection model. Vet Microbiol.

[24] Drolia R, Tenguria S, Durkes AC, Turner JR, Bhunia AK (2018). Listeria adhesion protein induces intestinal epithelial barrier dysfunction for bacterial translocation. Cell Host Microbe.

[25] Zhang J, Lu YC, Li SW, Ku XG, Liu XL, Memon AM, He QG, Bi DR, Meng XR (2018). Co-infection with porcine bocavirus and porcine circovirus 2 affects inflammatory cytokine production and tight junctions of IPEC-J2 cells. Virus Genes.

[26] Liu J, Zhang XL, Ma C, You JW, Dong M, Yun SF, Jiang P (2016). Heat shock protein 90 is essential for replication of porcine circovirus type 2 in PK-15 cells. Virus Res.

[27] Wang Y, Gagnon CA, Savard C, Music N, Srednik M, Segura M, Lachance C, Bellehumeur C, Gottschalk M (2013). Capsular sialic acid of Streptococcus suis serotype 2 binds to swine influenza virus and enhances bacterial interactions with virus-infected tracheal epithelial cells. Infect Immun.

[28] Livak KJ, Schmittgen TD (2001). Analysis of relative gene expression data using real-time quantitative PCR and the 2(T)(− ΔΔ C) method. Methods.

[29] Carrozzino F, Pugnale P, Feraille E, Montesano R (2009). Inhibition of basal p38 or JNK activity enhances epithelial barrier function through differential modulation of claudin expression. Am J Physiol Cell Physiol.

[30] Srinivasan B, Kolli AR, Esch MB, Abaci HE, Shuler ML, Hickman JJ (2015). TEER measurement techniques for in vitro barrier model systems. J Lab Autom.

[31] Chelakkot C, Ghim J, Ryu SH (2018). Mechanisms regulating intestinal barrier integrity and its pathological implications. Exp Mol Med.

[32] Kojima T, Go M, Takano K, Kurose M, Ohkuni T, Koizumi J, Kamekura R, Ogasawara N, Masaki T, Fuchimoto J, Obata K, Hirakawa S, Nomura K, Keira T, Miyata R, Fujii N, Tsutsumi H, Himi T, Sawada N (2013). Regulation of tight junctions in upper airway epithelium. Biomed Res Int.

[33] Sajjan U, Wang Q, Zhao Y, Gruenert DC, Hershenson MB (2008). Rhinovirus disrupts the barrier function of polarized airway epithelial cells. Am J Respir Crit Care Med.

[34] Nazli A, Chan O, Dobson-Belaire WN, Ouellet M, Tremblay MJ, Gray-Owen SD, Arsenault AL, Kaushic C (2010). Exposure to HIV-1 directly impairs mucosal epithelial barrier integrity allowing microbial translocation. PLoS Pathog.

[35] Misinzo G, Delputte PL, Lefebvre DJ, Nauwynck HJ (2009). Porcine circovirus 2 infection of epithelial cells is clathrin-, caveolae- and dynamin-independent, actin and Rho-GTPase-mediated, and enhanced by cholesterol depletion. Virus Res.

[36] Misinzo G, Delputte PL, Nauwynck HJ (2008). Inhibition of endosome-lysosome system acidification enhances porcine circovirus 2 infection of porcine epithelial cells. J Virol.

[37] Yan M, Zhu L, Yang Q (2014). Infection of porcine circovirus 2 (PCV2) in intestinal porcine epithelial cell line (IPEC-J2) and interaction between PCV2 and IPEC-J2 microfilaments. Virol J.

[38] Tenenbaum T, Matalon D, Adam R, Seibt A, Wewer C, Schwerk C, Galla HJ, Schroten H (2008). Dexamethasone prevents alteration of tight junction-associated proteins and barrier function in porcine choroid plexus epithelial cells after infection with Streptococcus suis in vitro. Brain Res.

[39] Naydenov NG, Hopkins AM, Ivanov AI (2009). c-Jun N-terminal kinase mediates disassembly of apical junctions in model intestinal epithelia. Cell Cycle.

[40] Konno T, Ninomiya T, Kohno T, Kikuchi S, Sawada N, Kojima T (2015). c-Jun N-terminal kinase inhibitor SP600125 enhances barrier function and elongation of human pancreatic cancer cell line HPAC in a Ca-switch model. Histochem Cell Biol.

[41] Lin Z, Zhang YG, Xia Y, Xu X, Jiao X, Sun J (2016). Salmonella enteritidis effector AvrA stabilizes intestinal tight junctions via the JNK pathway. J Biol Chem.

[42] Al-Sadi R, Guo S, Ye D, Dokladny K, Alhmoud T, Ereifej L, Said HM, Ma TY (2013). Mechanism of IL-1beta modulation of intestinal epithelial barrier involves p38 kinase and activating transcription factor-2 activation. J Immunol.

[43] Al-Sadi R, Guo SH, Ye DM, Ma TY (2013). TNF-alpha modulation of intestinal epithelial tight junction barrier is regulated by ERK1/2 activation of Elk-1. Am J Pathol.

[44] Zhang Y, Wu S, Ma J, Xia Y, Ai X, Sun J (2015). Bacterial protein AvrA stabilizes intestinal epithelial tight junctions via blockage of the C-Jun N-terminal kinase pathway. Tissue Barriers.

[45] Xiao Z, Liu L, Tao W, Pei X, Wang G, Wang M (2018). *Clostridium tyrobutyricum* protect intestinal barrier function from LPS-induced apoptosis via P38/JNK signaling pathway in IPEC-J2 Cells. Cell Physiol Biochem.

