# Supplementary figures and images for: Porcine circovirus type 2 exploits JNK-mediated disruption of tight junctions to facilitate Streptococcus suis translocation across the tracheal epithelium
Source: Vet Res. 2020 Feb 27;51:31. doi: 10.1186/s13567-020-00756-2 (PMC7047418; doi:10.1186/s13567-020-00756-2)

**ZO-1 (control)**

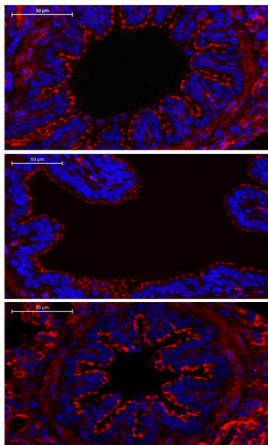

**ZO-1 (PCV2)**

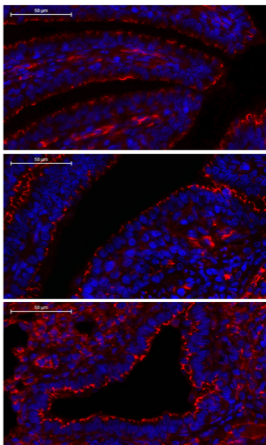

**occludin (control)**

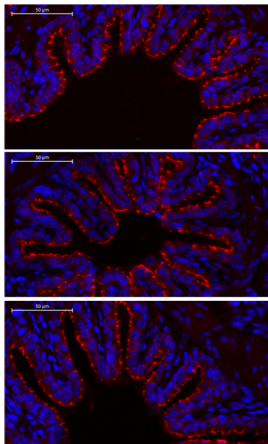

**occludin (PCV2)**

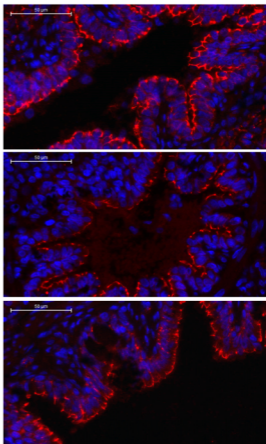

Supplement: Supplementary file 1 — Additional file 1. Immunofluorescence staining of ZO-1 and occludin in the lungs. [file 13567_2020_756_MOESM1_ESM.pdf]
