# Supplementary material for: Porcine circovirus type 2 exploits JNK-mediated disruption of tight junctions to facilitate Streptococcus suis translocation across the tracheal epithelium
Source: Vet Res. 2020 Feb 27;51:31. doi: 10.1186/s13567-020-00756-2 (PMC7047418; doi:10.1186/s13567-020-00756-2)
Supplement: Supplementary file 2 — Additional file 2. CFU number of adherent bacteria to STEC per well. The results are shown as mean ± SD of five independent experiments. [file 13567_2020_756_MOESM2_ESM.docx]

Additional file 2 CFU number of adherent bacteria to STEC per well.

| Infection time of PCV2 | SS2 | PCV2+SS2 |
| --- | --- | --- |
| 24 h | (54.450 ± 7.623) × 10^4^ | (31.200 ± 4.329) × 10^4^ |
| 36 h | (58.225 ± 4.448) × 10^4^ | (40.350 ± 3.687) × 10^4^ |
| 48 h | (43.200 ± 2.514) × 10^4^ | (51.300 ± 6.433) × 10^4^ |

The results were shown as Mean ± SD of five independent experiments.
