# Supplementary material for: Porcine circovirus type 2 exploits JNK-mediated disruption of tight junctions to facilitate Streptococcus suis translocation across the tracheal epithelium
Source: Vet Res. 2020 Feb 27;51:31. doi: 10.1186/s13567-020-00756-2 (PMC7047418; doi:10.1186/s13567-020-00756-2)
Supplement: Supplementary file 3 — Additional file 3. CFU number of invasive bacteria to STEC per well. The results are shown as mean ± SD of five independent experiments. [file 13567_2020_756_MOESM3_ESM.docx]

Additional file 3 CFU number of invasive bacteria to STEC per well.

| Infection time of PCV2 | SS2 | PCV2+SS2 |
| --- | --- | --- |
| 24 h | (15.534 ± 5.301) × 10^3^ | (7.896 ± 0.980) × 10^3^ |
| 36 h | (11.416 ± 2.791) × 10^3^ | (11.22 ± 2.059) × 10^3^ |
| 48 h | (10.324 ± 2.060) × 10^3^ | (8.708 ± 3.828) × 10^3^ |

The results were shown as Mean ± SD of five independent experiments.
